# HS1 Is Involved in Hygromycin Resistance Through Facilitating Hygromycin Phosphotransferase Transportation From Cytosol to Chloroplast

**DOI:** 10.3389/fpls.2020.00613

**Published:** 2020-05-27

**Authors:** Yanzhong Luo, Lan Zhang, Weiwei Li, Miaoyun Xu, Chunyi Zhang, Lei Wang

**Affiliations:** Biotechnology Research Institute, Chinese Academy of Agricultural Sciences, Beijing, China

**Keywords:** *Arabidopsis thaliana*, chloroplast, HS1, HPT, transit peptide-less protein, transportation, hygromycin sensitivity

## Abstract

The transportation of proteins encoded by nuclear genes from plant cytosol to chloroplast is essential for chloroplast functions. Proteins that have a chloroplast transit peptide (cTP) are imported into chloroplasts via translocases on the outer and inner chloroplast envelope. How proteins lacking transit sequence are imported into chloroplast remains largely unknown. During screening of an Arabidopsis population transformed with a hairpin RNA gene-silencing library, we identified some transgenic plants that had active expression of the selectable marker gene, hygromycin phosphotransferase (HPT), but were sensitive to the selection agent, hygromycin B (HyB). Mutant and complementation analysis showed that this HyB sensitivity of transgenic plants was due to silencing of the HS1 (Hygromycin-Sensitive 1) gene. HS1 is localized in the chloroplast and interacts physically with HPT in yeast cells and *in planta*. Fluorescence and immunoblotting analysis showed that HPT could not be transported effectively into chloroplasts in *Aths1*, which resulted in *Aths1* is sensitivity to hygromycin on higher HyB-containing medium. These data revealed that HS1 is involved in HyB resistance in transgenic Arabidopsis through facilitating cytosol-chloroplast transportation of HPT. Our findings provide novel insights on transportation of chloroplast cTP-less proteins.

## Introduction

Chloroplasts are essential organelles of endosymbiotic origin in plants and function in photosynthesis and other important physiological processes (Whatley, 1978; Nelson and Ben-Shem, 2004; Reinbothe et al., 2010). Chloroplasts are semiautonomous organelles in plant cells and contain c. 3,000–4,000 proteins. Of these proteins, only 50–200 are encoded in the organellar genome; therefore more than 95% of chloroplasts proteins need to be imported from the cytosol (Keegstra and Cline, 1999; Cline and Dabney-Smith, 2008; Kessler and Schnell, 2009; Rolland et al., 2012). Nuclear-encoded proteins are synthesized on cytoplasmic ribosomes and are imported and post-translationally translocated across chloroplast envelope membranes. Hence active transport of chloroplast proteins is essential for chloroplast function.

Few studies have investigated in detail the distinctive routing and import systems of preproteins targeted to chloroplasts. The majority of nucleus-encoded plastid proteins are targeted to chloroplasts through sequential interactions between their NH_2_-terminal transit peptides and translocons at the outer and inner envelope membranes (Li and Chiu, 2010; Shi and Theg, 2013). The transport of nucleus-encoded plastid proteins require the core translocon at the outer envelope membrane of chloroplast (TOC) complex formed by TOC159, TOC75 and TOC34, and chloroplast translocon at the inner envelop membrane of chloroplast (TIC) complex such as TIC110, TIC20, TIC21, and TIC236. These complexes are suggested to function as a translocation channel for preproteins (Inaba et al., 2003; Soll and Schleiff, 2004; Inaba et al., 2005; Kessler and Schnell, 2009; Li and Chiu, 2010; Chen et al., 2018). Second, proteins on the chloroplast outer envelope membrane are targeted through a cytosolic sorting system, which prevents mistargeting of these proteins to the outer membrane of other organelles such as the endoplasmic reticulum (ER) or mitochondria (Lee et al., 2013; Li et al., 2017). During this process, cytosolic factors such as Hsp70 and 14-3-3 proteins act to facilitate the passage of precursors from the cytosol to chloroplasts (May and Soll, 2000; Hiltbrunner et al., 2001; Zhang and Glaser, 2002). Third, the proteins initially targeted to the secretion (Sec) translocon at the ER/Golgi apparatus and co-translational proteins are subsequently delivered to the chloroplast via vesicle trafficking or other unknown pathways (Chen et al., 2004; Villarejo et al., 2005; Nanjo et al., 2006; Radhamony and Theg, 2006), which may be a vestige of an early endosymbiotic event; Finally, a set of proteins that lack cleavable targeting signals are imported into chloroplast by a largely uncharacterized system (Kleffmann et al., 2004; Nada and Soll, 2004; Miras et al., 2007).

As mentioned above, the TOC/TIC translocon system is the general and constitutes major protein import pathway into chloroplasts. Chloroplast proteomic data showed that about 30% of chloroplast-detected proteins did not possess identifiable transit peptides (Kleffmann et al., 2004). It has been reported that some of these proteins such as amylase and glycosylated plastid proteins are transported into chloroplasts via vesicle trafficking or by other unknown mechanisms (Chen et al., 2004; Kleffmann et al., 2004; Nada and Soll, 2004; Asatsuma et al., 2005; Villarejo et al., 2005; Nanjo et al., 2006; Miras et al., 2007; Kitajima et al., 2009). However, the details of the process remain unknown. Furthermore, it is largely unknown how proteins lacking transit peptides are imported into chloroplasts.

Hygromycin B (HyB) is an aminoglycoside antibiotic produced by *Streptomyces hygroscopicus* that primarily inhibits protein synthesis both in prokaryotic and eukaryotic cells (Davies et al., 1965; Gonzalez et al., 1978; Eustice and Wilhelm, 1984; McGaha and Champney, 2007). In addition to inhibiting protein synthesis in the cytosol (Gonzalez et al., 1978), HyB also affect protein synthesis of the mitochondria and chloroplast (Moazed and Noller, 1987; Brodersen et al., 2000). An *Escherichia coli* originated hygromycin phosphotransferase (HPT, E.C. 2.7.1.119) can inactivate HyB by adding a phosphate group to position seven of the destomic acid ring of HyB both *in vitro* and *in vivo* (Pardo et al., 1985). HPT has therefore been widely used as a selectable marker in plant transformation (Miki and McHugh, 2004). It was reported that HPT was present both in the cytoplasm and the extracelluar space in HPT-transgenic plants (Zhang et al., 2011). However, few studies have elucidated the relationship between plant responses to HyB and the distribution of HPT in subcellular organelles.

In this study, we screened a transgenic *Arabidopsis* population transformed with a library of long hairpin RNA (hpRNA) gene silencing constructs using HPT as the selective marker. We identified several HyB-sensitive transgenic lines in which HPT was expressed. We found that HPT was localized both in the cytosol and in chloroplasts. The phenotype of these plants sensitive to HyB was due to the silencing of an unknown gene we designated HS1. Further analysis demonstrated that HS1 could interact physically with the HPT protein, showing HS1 is involved in cytosol-chloroplast transport of the HPT protein.

## Materials and Methods

### Plant Materials and Growth Conditions

*Arabidopsis thaliana* (ecotype Columbia, Col-0) seedlings were grown on half-strength Murashige and Skoog (MS) solid medium supplied with different concentrations (15 mg/L, 25 mg/L, and 50 mg/L) of HyB for screening mutant transgenic plants at 22°C under 14-h light/10-h dark cycles. The plants used for protoplast and chloroplast preparation were cultured in soil for 1 month at 22°C under 12 h:12 h, light:dark cycles in a glasshouse.

### Identification of *HS1*RNAi, *hs1-1*, and *hs1-2* Mutants

The *HS1*RNAi mutants were isolated from the screening of an *Arabidopsis* RNAi library in medium containing 25 mg/L HyB. The sensitive plants were transferred from the medium and planted in a glasshouse. Total DNA was extracted from T1 plants and analyzed by PCR using P35S and Inrv4 primers.

T-DNA insertion lines of the HS1 gene, *hs1-1* and *hs1-2*, were ordered from the Arabidopsis Biological Resource Center (ABRC) (SALK_029285C and SALK_060883C), and their genotypes were confirmed by PCR analysis using a common primer, LBb1, and gene-specific primer pairs, hs1-1F, hs1-1R, hs1-2F, and hs1-2R. The nucleotide sequences of the primer pairs are listed in Supplementary Table S1.

### Arabidopsis Transformation and Phenotypic Analysis

To generate the binary *HS1*RNAi construct, the 117-bp fragment of the *HS1* coding sequence was amplified from the cDNA of WT *Arabidopsis* using the primer pair HS1RiF and HS1RiR, which generated inverted repeat sequences, using the RMHR protocol described previously (Wang et al., 2008). Inverted-repeats of *HS1* fragments were inserted into the *Xba* I-*Sac* I site in pCAMBIA1303-GUS, leading to replacement of the GUS cassette. Constructs were transformed into *Agrobacterium tumefaciens* strain GV3101 and then into *Arabidopsis* as described previously (Bent, 2000). All transgenic plants, from at least three transgenic lines each include *HS1-GUSGFP*, *GUSGFP*, *hs1-1*/*HPT*/*HS1*, *hs1-1*/*HPT*, *WT*/*HPT*, and *WT/HPT/HS1*, were used in the relevant experiment, and results of representative lines were used for qualitative analysis.

### Vector Construction

The pCAMBIA1303-GUS vector was generated by subcloning the GUS expression cassette of pBI121 (Chen et al., 2003) into the *Hind*III and *Eco*RI sites of pCAMBIA1303 (Hajdukiewicz et al., 1994). For complementation analysis, full length of *HS1* cDNA was inserted into *Xba* I-*Sac* I sites in pCAMBIA1303-GUS, generating the HS1 overexpression construct.

The other constructs used in this text were amplified by PCR and inserted into the corresponding vectors. Primer pairs used in this work are listed in Supplementary Tables S1, S2.

### Isolation of Intact Chloroplasts

Chloroplasts were isolated from *Arabidopsis* leaves using a chloroplast isolation kit (Sigma, CPISO-1KT) following the manufacturer’s instructions. Briefly, *Arabidopsis* leaves were harvested and homogenized in chloroplast isolation buffer (CIB). The homogenate was filtered through Miracloth (Sigma, 475855-1R). Homogenization and filtration were repeated twice. The suspension containing chloroplasts was sedimented by centrifugation at 4°C, 1,000 × *g* for 7 min. The pellets were placed in tubes with CIB and resuspended by gently pipetting up and down. The resuspended material was then loaded onto a 40% Percoll solution and centrifuged for 6 min at 1,700 × *g*. Intact chloroplasts were collected at the bottom as a small green pellet. Intact chloroplasts were collected and washed twice with CIB without BSA. The purified chloroplasts were visualized under a Leica light microscope (Leica). For immunoblotting analysis, chloroplasts were isolated from 10- to 14-day-old seedlings grown in half-strength MS plate medium. The contamination extent of chloroplast proteins was analyzed by western blot (Supplementary Figure S6).

### Protoplast Transient Expression Assay

Preparation and transformation of *Arabidopsis* mesophyll protoplasts were performed as described previously (Yoo et al., 2007). After overnight culture of transformed protoplasts, fluorescence from green fluorescent protein (GFP) in the protoplasts was examined using confocal microscopy (Carl Zeiss LSM510) under 488 nm excitation. The emission wavelength was restricted to 530 nm for green fluorescence and 650 nm for red fluorescence of the chlorophyll background.

### Protein Preparation and Immunoblotting Analysis

Total proteins were extracted from *Arabidopsis* leaves as described previously (Martínez-García et al., 1999). Chloroplast proteins were extracted as described by Fan et al. (2009). In short, proteins were extracted using an extraction buffer (500 mM Tris-HCl, pH 7.5, 150 mM NaCl, 0.1% NP-40, 4 M urea, and 1 mM phenylmethanesulfonyl fluoride). After centrifugation at 13,000 × *g* for 10 min at 4°C, the supernatant was transferred into a new tube and quantified using the Bio-Rad DC protein assay (Bio-Rad). For immunoblotting analysis, proteins were separated by SDS-PAGE and transferred to polyvinylidene difluoride (PVDF) membranes. The membranes were incubated with specific primary antibodies, and signals from secondary horseradish peroxidase conjugated antibodies were detected using enhanced chemiluminescence according to the manufacturer’s protocol.

### Co-immunoprecipitation

Total proteins extracted from 10-day-old seedlings were immunoprecipitated using anti-GFP as previously described (Speth et al., 2014). After washing the Sepharose G beads five times, the bound proteins were eluted and separated on SDS-PAGE gels. Immunoblotting was performed using anti-HPT (Beijing Protein Innovation Co., Ltd.) and anti-ACTIN (Abmart) monoclonal antibodies.

### Yeast Two-Hybrid Assays

The Matchmaker Gold Yeast Two-Hybrid System from Clontech was used for Gal4-based yeast two-hybrid assays according to the manufacturer’s instructions. For all experiments, we used the yeast host strain AH109. The prey and bait plasmids were constructed using the pGADT7 and pGBKT7 vectors, respectively. The plasmids constructed for yeast assays are listed under “Vector construction.” The prey and bait constructs were simultaneously co-transformed into AH109 and plated on synthetic dropout (SD) medium without tryptophan or leucine (SD/−Leu/−Trp) and grown at 30°C for 4 days. The grown cells were cultured on SD/−Leu/−Trp liquid medium to an OD_600_ ≈ 0.8. Equivalent numbers of co-transformed yeast cells (normalized based on absorbance) were serially diluted 10-fold, and 10 μL of each dilution were plated on SD medium without tryptophan and leucine (SD/−Leu/−Trp), and on SD medium without tryptophan, leucine, histidine, and adenine and supplemented with 20 mg/L X-α-Gal (SD/−His/−Leu/−Trp/−Ade/+X-α-Gal).

### Immunogold Labeling of Ultrathin Sections

Immunogold labeling was performed following a modified protocol described by Wilson and Bacic (2012). In brief, 10-day-old seedling leaves were fixed in 0.1 M PBS buffer containing 4% (v/v) paraformaldehyde and 0.25% (v/v) glutaraldehyde for 4 h at 4°C. To prevent antigenicity, osmium tetroxide was not used in the fixative solution. After washing with phosphate buffer and dehydration through a gradient of ethanol at 4°C, samples were embedded in resin (LR White, Britain). Ultrathin sections were prepared with an EM UC7 microtome (Leica, Germany) and collected on nickel grids. After being moistened in double-distilled water for 5 min, the ultrathin sections on nickel grids were blocked in blocking buffer (0.12% glycine in PBS) for 20 min. The nickel grids were subsequently incubated with 1:500 diluted anti-HPT monoclonal antibody (Beijing Protein Innovation Co., Ltd.) in blocking buffer for 12 h. After washing, the nickel grids were incubated with 1:20 diluted colloidal gold-conjugated goat anti-mouse immunoglobulin G (Sigma-Aldrich) in blocking buffer for 1.5 h. The nickel grids were then placed in a clear incubation chamber and each slide was covered with freshly prepared silver enhancement solution for 10 min at ∼20°C to allow growth of gold particles. To investigate the specificity of immuno-labeling, a control experiment was conducted after omission of the primary incubation stage with HPT antibody. Immunized ultrathin sections were washed, post-stained, and examined with a transmission electron microscope (Hitachi H-7650, Japan).

## Results

### Identification of Hygromycin B-Sensitive Mutants

**FIGURE 1:**
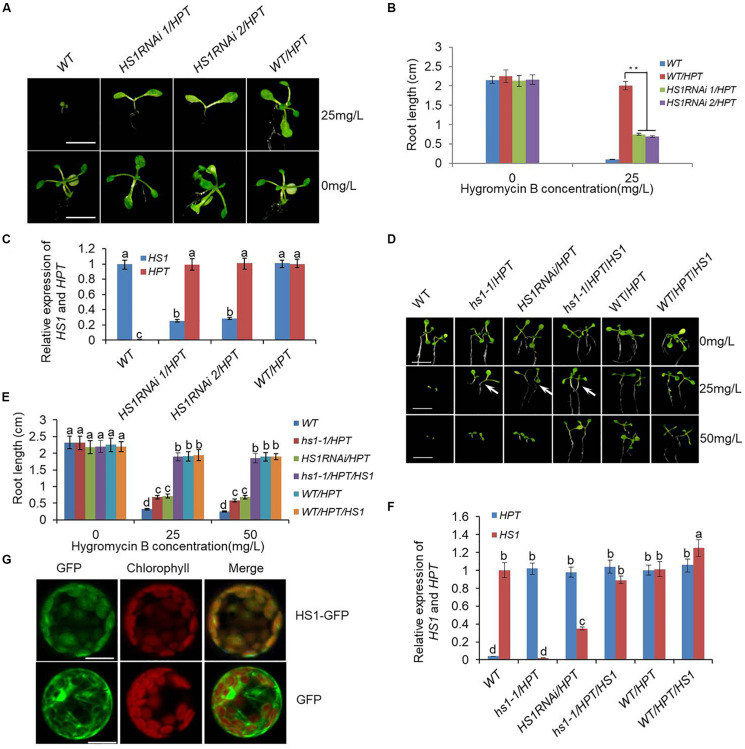
Genetic identification and localization of HS1. **(A)** Phenotype of *HS1*RNAi/*HPT* mutants. Ten-day-old seedlings of the wild type (WT), *HS1*RNAi/*HPT* mutant lines, and empty vector transgenic lines used as the control (WT/*HPT*) were grown on 0.5× MS medium supplemented with or without HyB as indicated on the right. *HS1*RNAi/*HPT* mutants show a phenotypic defect characterized by the absence of true leaves and repressed root development in HyB-containing medium. In contrast, *HS1*RNAi/*HPT* mutant growth was almost normal in medium without HyB. Bars = 3 mm. **(B)** Root length measurement of 10-day-old seedlings in **(A)** grown in the presence and absence of HyB-containing medium as indicated. Values are means ± SD of 30–40 seedlings from each transgenic line (**P < 0.01). **(C)** Quantitative real-time PCR analysis of *HS1* and *HPT* using *ACTIN* as a reference. T2 generation 10-day-old seedlings and original *HS1*RNAi/*HPT* lines screened from the hpRNA mutant library were used for analysis. Data represent mean ± SD of three replications. **(D)** *HS1* constitutive expression rescues the phenotypic defect of *HS1* mutants grown in HyB-containing medium. Seeds of plants indicated on the top of each panel were germinated on 0.5× MS medium without or with different concentrations of HyB as indicated on the right and grown for 10 days. *hs1-1*/*HPT* shows the same phenotypic defect as *HS1*RNAi mutants (*HS1*RNAi/*HPT*) in plates containing 25 mg/L HyB, while *hs1-1*/*HPT* complementary transgenic plants (*hs1-1*/*HPT*/*HS1*), transgenic control (WT/*HPT*), and overexpression plants (WT/*HPT*/*HS1*) grew normally. Bars = 5 mm. **(E)** Measurement of seedling root length is the same as described in **(D)**. Values are means ± SD of 40–50 seedlings of representative transgenic lines from three independent experiments. **(F)** Quantitative PCR analysis of *HS1* and *HPT* using *ACTIN* as a reference. Analysis of 10-day-old seedlings of re-transformed *HS1* hpRNA lines and other plants are as indicated on the *x*-axis. Data represent mean ± SD of three replications. **(G)** Subcellular localization of HS1 by GFP fluorescence. HS1-GFP fluorescence shows HS1 is localized in chloroplasts. Green indicates GFP fluorescence, red shows chloroplast autofluorescence, and yellow shows co-localization in the merged images. The constructs used for transformation are indicated at the right: GFP, as non-fusion GFP protein control; HS1-GFP, signal from the HS1-GFP fusion protein. Bar = 10 μm. For each column in **(C)**, **(E)**, and **(F)**, different letters *a*, *b*, *c*, *d* indicates significant differences at *P* < 0.05.

We previously developed a rolling-circle amplification-based method for constructing libraries of long hpRNA gene-silencing constructs targeting all expressed genes (Wang et al., 2008), and obtained over 5,000 hpRNA transgenic *Arabidopsis* lines using HyB as the selective agent. These transgenic lines exhibited various phenotypes associated with the silencing of various target genes (Supplementary Figure S1). Several of the hpRNA lines showed a distinct phenotype: they grew green cotyledons, but were unable to grow true leaves on MS medium containing 25 mg/L HyB. Furthermore, the phenotypic defective plants were able to complete their life cycle when transferred from a HyB-containing plate to soil. Once in HyB-free MS medium, these plants grew normally. Therefore, these plants differed from untransformed plants, which were dwarfed on the MS-HyB medium after 10 days, and normal transgenic lines, which grew vigorously under high concentrations of HyB (Figures 1A,B). These results indicated that these hpRNA lines had very low levels of HyB resistance, based on stunted growth of seedlings. Surprisingly, RNA expression analysis showed that the selectable marker gene HPT, which confers HyB resistance, was expressed at similar levels in HyB-sensitive plants and HyB-resistant plants, as measured by quantitative real-time PCR (Figure 1C). This indicated that HyB sensitivity in these plants was not due to lack of HPT expression. We then surmised that the endogenous gene(s) targeted by the hpRNA construct(s) in these transgenic lines must have been responsible for the phenotype. PCR amplification and sequencing of the hpRNA transgenes in four of the HyB-sensitive plants showed that the same endogenous gene, named *HS1*, was targeted in all four transgenic lines. We further confirmed that *HS1* was silenced by the hpRNA construct in these HyB-sensitive plants (referred to as *HS1*RNAi plants hereafter) (Figure 1C).

### HS1 Is Required for HyB Resistance in Transgenic Plants

The original *HS1*RNAi lines were obtained from transformation of *Arabidopsis* with an hpRNA library containing mixed constructs targeting all expressed genes. To confirm that the silencing of *HS1* was responsible for HyB sensitivity, *Arabidopsis* was transformed with a single *HS1* hpRNA construct to specifically silence the *HS1* gene. The re-transformed plants consistently displayed a HyB-sensitive phenotype while showing high-level HPT gene expression (Supplementary Figure S2).

To further demonstrate that *HS1* silencing was responsible for HyB sensitivity, we obtained two T-DNA insertion lines, *hs1-1* and *hs1-2*, in which *HS1* expression was either lost due to T-DNA insertion into the exon region (*hs1-1*) or downregulated due to T-DNA insertion into the promoter (*hs1-2*) (Supplementary Figure S3). When the *HPT* gene in empty vector was transformed into *hs1-1*, the resulting transgenic plants displayed HyB-sensitive phenotypes similar to the *HS1*RNAi plants, in contrast to *HPT* transgenic plants of wild-type background which showed strong HyB resistance (Figures 1D,E). This occurred despite *HPT* expression levels being similar between the two transgenic populations (Figure 1F). To further confirm the role of *HS1* silencing on HyB sensitivity, we transformed *hs1-1*/*HPT* lines, which were sensitive to HyB, with a construct expressing WT *HS1* for complementation, and assayed the resulting transgenic plants for HyB resistance. As shown in Figures 1D–F, the introduction of a functional *HS1* gene made the *hs1-1*/*HPT/HS1* transgenic plants resistant to HyB, confirming that mutation of *HS1* is responsible for the phenotype defect and *HS1* is required for HyB resistance.

### HS1 Localizes in the Chloroplast

*HS1* (At5g55210) encodes a previously unknown protein consisting of 168 amino acids. ChloroP program analysis suggested that HS1 contains a putative chloroplast transit peptide (cTP) in the *N*-terminal region (residues 1–80) (Emanuelsson et al., 1999). A GenBank database search^1^ revealed dozens of proteins that were highly homologous to HS1 in diverse higher plant species (Supplementary Figures S4, S5), indicating that the function of HS1 and its homologs may be conserved. Even though HS1 is highly conserved among higher plants, domain sequence analysis did not provide any clues about its biochemical roles. To gain insight into the function of HS1, we first investigated the subcellular localization of HS1 using an HS1 construct with GFP fused to the carboxy terminus (HS1-GFP). This construct was expressed transiently in Arabidopsis protoplasts. GFP fluorescence was colocalized exclusively with chloroplastic chlorophyll, whereas that from the GFP control did not (Figure 1G), indicating that HS1 is indeed a chloroplast-targeted protein.

### HS1 Is Involved in Cytosol to Chloroplast Transport of HPT

**FIGURE 2:**
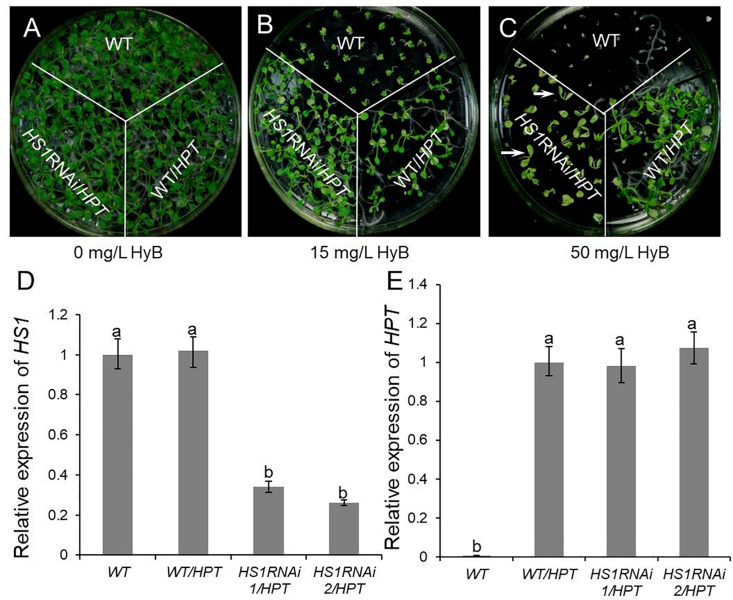
Knockdown of *HS1* increases the sensitivity to hygromycin B in transgenic plants. Ten days seedlings of WT (Col-0), *HS1*RNAi mutants, and empty vector transgenic control (WT/*HPT*) respond to hygromycin in 0 mg/L **(A)**,15 mg/L **(B)**, and 50 mg/L **(C)** containing plates. All plants had normal growth on medium without hygromycin B. *HS1*RNAi/*HPT* plants grew similar to empty vector transgenic plants on 15 mg/L hygromycin B medium, whereas growth of the WT was repressed. *HS1*RNAi/*HPT* plants (arrowhead) did not grow true leaves on medium with 50 mg/L hygromycin B, whereas transgenic control plants grew normally. **(D)** and **(E)** are quantitative PCR expression analysis of *HS1* **(D)** and *HPT* **(E)** using *ACTIN* as reference in the WT, *HS1*RNAi/*HPT* mutants, and empty vector transgenic plants which grew on 0.5× MS medium without hygromycin. Although *HS1* expression was silenced in *HS1*RNAi/*HPT* plants, *HPT* expression was similar in transgenic control and RNAi mutants. Similar results were obtained in three independent experiments. For each column in **(D)** and **(E)**, different letters *a*, *b* indicates significant differences at *P* < 0.05.

The extent of the phenotypic defect varied with the concentration of HyB (Figure 1D). Specifically, the growth of *HS1* RNAi mutants was similar to that of plants transformed with empty vector in plates with low HyB concentration (15 mg/L), and better than WT (Figures 2A–C). However, the growth of *HS1* RNAi plants was repressed significantly in plates with high HyB concentration (50 mg/L), whereas empty vector-transformed plants grew well. Transcripts of *HS1* were decreased significantly in *HS1* RNAi mutant plants compared with those of the transgenic control, although *HPT* expression was similar in *HS1* mutants and control plants (Figures 1F, 2D,E).

**FIGURE 3:**
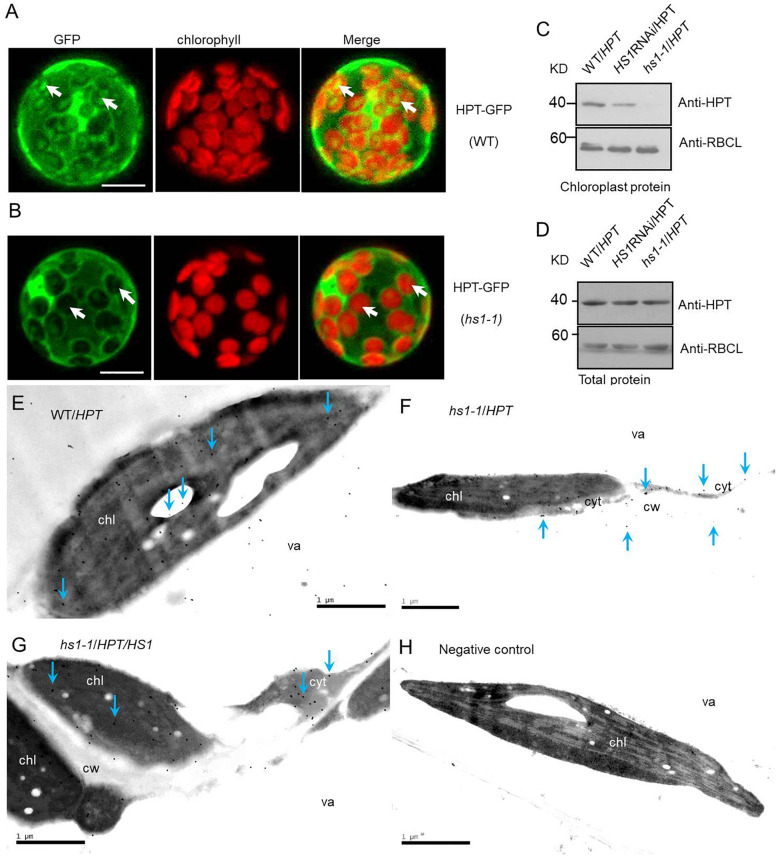
HS1 is involved in cytosol-to-chloroplast transport of HPT. Subcellular localization of HPT-GFP in the WT **(A)** and *hs1-1* **(B)** backgrounds in Arabidopsis. HPT-GFP constructs were transformed transiently into plant cells. Arrowheads indicate higher chloroplast GFP fluorescence intensity in a mesophyll protoplast of WT compared to *hs1-1*. Bars = 10 μm. **(C**,**D)** Western blot of HPT in WT/*HPT* (empty vector transgenic lines), *HS1*RNAi/*HPT*, and *hs1-1*/*HPT* (empty vector transformed into *hs1-1*) transgenic plants. HPT accumulation was reduced significantly or undetectable in *HS1*RNAi/*HPT* and *hs1-1/HPT* chloroplasts compared with the control **(C)**, while total HPT varied slightly **(D)**. The large subunit of RUBISCO (RBCL) was probed as a protein loading control. Approximately 20 μg **(C)** and 10 μg **(D)** of protein were loaded in each lane. Similar results were obtained in two additional independent experiments. **(E–H)** Transmission electron microscopic images of immunogold localization of HPT in transgenic *Arabidopsis*. HPT in ultrathin leaf sections reacted with anti-HPT antibody and a gold-conjugated secondary antibody. Gold particles were detected by transmission electron microscopy. Immunogold labeling of HPT in leaves from WT/*HPT* **(E)**, *hs1-1*/*HPT* **(F)**, *hs1-1*/*HPT*/*HS1* **(G)**, negative control **(H)** transgenic *Arabidopsis* plants, and negative control without anti-HPT antibody in blocking buffer showed no gold particles. Arrowheads show typical gold particles (10 nm, black dots). HPT present inside of chloroplasts (chl, the dark area) in *WT*/*HPT*, *hs1-1*/*HPT*/*HS1*, and outside of chloroplasts in *hs1-1*/*HPT*. Three different leaf samples and more than 30 immunogold-labeled positive cells were observed with similar results. Images represent typical observations in different leaf samples. chl, chloroplast; cyt, cytosol; va, vacuole; cw, cell wall. Bars = 1 μm.

Hygromycin phosphotransferase expression accompanied by the absence of HyB resistance in *HS1*-silenced plants, plus chloroplast localization of HS1, led us to surmise that HPT may function in chloroplasts in addition to cytoplasm. Moreover, HS1 may play a role in chloroplast import of HPT. To test whether HS1 is involved in transporting HPT to the chloroplast, we first examined the transient expression of an HPT-GFP fusion protein in Arabidopsis mesophyll protoplasts in plants of WT and *hs1-1* background. As shown in Figure 3A, the accumulation of HPT-GFP fusion protein, as indicated by GFP fluorescence, was detected in the cytosol and chloroplasts of transformed WT protoplasts, while GFP fluorescence was almost undetectable in chloroplasts of transformed *hs1-1* protoplasts (Figure 3B). To further examine amount change of HPT protein in plants of different genetic background, Western blot analysis of protein in HyB-resistant transgenic plants were performed. HPT accumulation was examined in total protein and chloroplast protein extracts. As shown in Figures 3C,D, HPT was present in total protein and chloroplast protein extracts in *WT*/*HPT* transgenic plants. While the total amount of HPT protein was similar among *WT*/*HPT*, *HS1*RNAi/*HPT*, and *hs1-1*/*HPT* transgenic plants (Figure 3D), the amount of chloroplast HPT protein was reduced in *HS1*RNAi/*HPT* plants compared with *WT*/*HPT* and was almost below detection in *hs1-1*/*HPT* plants (Figures 3C,D). These results suggest that HPT can be imported into chloroplasts even though it lacks a chloroplast-targeting cTP sequence.

To further investigate if HS1 is required for HPT transport into chloroplasts, immunogold labeling and transmission electron microscopy were performed. As shown in Figures 3E–H, HPT also targets to chloroplast in addition to localizing in the cytosol. Furthermore, the amount of chloroplast HPT was reduced drastically in *hs1-1*/*HPT* compared with *WT/HPT* and complementary transgenic *hs1-1*/*HPT*/*HS1*. Taken together, these results suggest that reduced transport of HPT to chloroplasts contributes to the phenotypic defects observed in *HS1* mutants and that HS1 regulates HPT entry into chloroplasts and is required for HPT transport from the cytosol to chloroplasts.

### HS1 Physically Interacts With HPT

**FIGURE 4:**
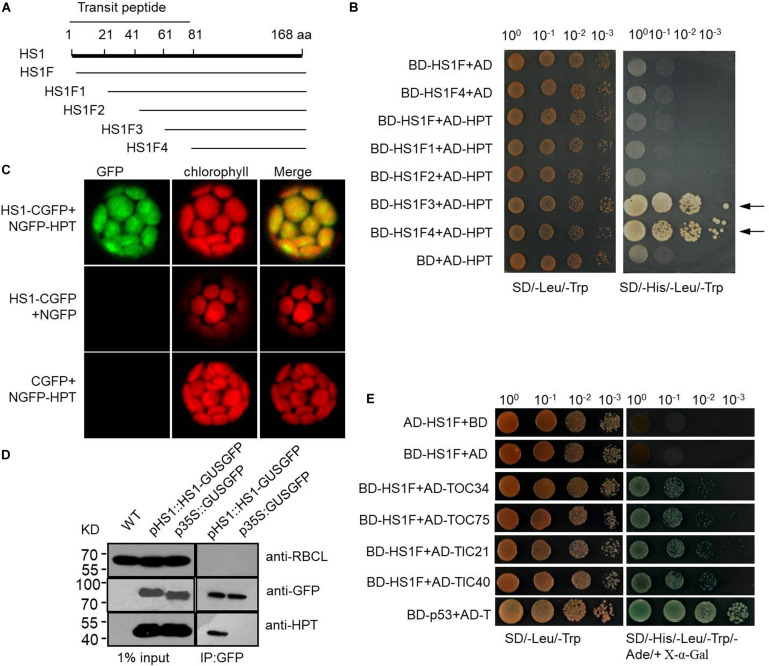
HS1 interacts physically with HPT and with endogenous chloroplast proteins. **(A)** Schematic of HS1 proteins truncated from the *N*-terminus every 20 amino acids. HS1F1–F4 has *N*-terminal deletions of HS1 as shown. **(B)** Yeast two-hybrid assay showing HS1 interaction with HPT. Plasmid constructs used for co-transformation are indicated on the left. **(C)** Bimolecular fluorescence complementation (BiFC) assay. HS1 and HPT were fused to NGFP and CGFP (NGFP and CGFP are *N*-terminal and *C*-terminal fragments, respectively, from split GFP) and transiently co-transformed into plant cells. GFP fluorescence was detected in chloroplasts (top). **(D)** Co-immunoprecipitation assay showing interaction between HPT and HS1. *Arabidopsis* total proteins were extracted from 10-day-old WT and transgenic *Arabidopsis* seedlings. Proteins were first immunoprecipitated with anti-GFP antibody then analyzed by immunoblot with antibody against HPT. HPT was detected only in the IP products from HS-GUSGFP, but not GUSGFP plants. The large subunit of RUBISCO (RBCL) and GFP were probed as controls. Constructs used to transform plants are indicated on the top of each panel. **(E)** HS1 interacts with TOC/TIC complex proteins. Various proteins with AD fusion are used in a yeast two-hybrid assay with BD-HS1F (Figure 4A) as the bait, four proteins from the TOC/TIC complex (TOC34, TOC75, TIC 21, and TIC 40) were fused to AD. The BD-HS1F + AD combination is used as a negative control, and interaction of BD-p53 and AD-T served as the positive control. Equivalent numbers of co-transformed yeast cells were serially diluted 10-fold and plated on SD/–Leu/–Trp medium and on SD/–His/–Leu/–Trp/–Ade/+X-α-Gal medium, respectively. Locus encoded selected proteins as follows: HS1 (AT5G55210), TOC75 (AT3G46740), TIC40 (AT5G16620), TOC34 (AT5G05000), and TIC21 (AT2G15290).

One possible way for HS1 to promote the import of HPT is by physically interacting with HPT and guiding the protein to the chloroplast. To examine this possibility, we performed yeast two-hybrid assays using HPT fused to the BD domain of GAL4 and a series of *N*-terminus-truncated HS1 sequences fused to the AD domain of GAL4 (Figure 4A). Among the truncated sequences assayed, HS1F3 and HS1F4 showed strong interaction with HPT, as indicated by the growth of yeast cells co-transformed with BD-HS1F3 and AD-HPT or BD-HS1F4 and AD-HPT constructs (Figure 4B). Under the influence of the transit peptide of HS1, full-length and partially truncated HS1 may not be imported into nuclei and does not show interaction with HPT. This indicated that the *C*-terminus of HS1 could interact with HPT.

We also performed a bimolecular fluorescence complementation (BiFC) assay to further examine the interaction between HS1 and HPT. The *N*-terminus (aa 1–177) of GFP (NGFP) was fused with HS1, and the *C*-terminus (aa 178-238) of GFP (CGFP) was fused with HPT. When NGFP-HS1 and HPT-CGFP constructs were co-transformed into *Arabidopsis* protoplasts, GFP fluorescence was detected in chloroplasts (Figure 4C), indicating that HS1 interacted directly with HPT in chloroplasts. Furthermore, we used immunoprecipitation (IP) with anti-GFP antibody to pull-down proteins from plants transformed with HS1-GUSGFP or GUSGFP constructs, and then examined the presence of HPT in the IP product by western blot analysis. As shown in Figure 4D, HPT was detected from the IP pull-down product of HS1-GUSGFP transformed plants but not GUSGFP-transformed plants, again indicating that HS1 binds directly to HPT proteins in *Arabidopsis* cells. Taken together, these results suggest that HS1 physically interacts with HPT, and this interaction is involved in chloroplast import of HPT.

### HS1 Can Interact With TOC/TIC Complex Proteins

HS1 itself has an identifiable chloroplast targeting signal, indicating that HS1 is likely to transport across the chloroplast envelope through the classical TOC/TIC complex. To verify this speculation, yeast two-hybrid assays were used to determine the interaction between HS1 and TOC/TIC complex proteins (Figure 4E). The data showed HS1 can interact with TOC34, TOC75, TIC21, and TIC40 proteins, indicating that HS1 is likely to mediate transport of HPT (without predictable cTP) into chloroplasts via the classical TOC/TIC pathway.

## Discussion

**FIGURE 5:**
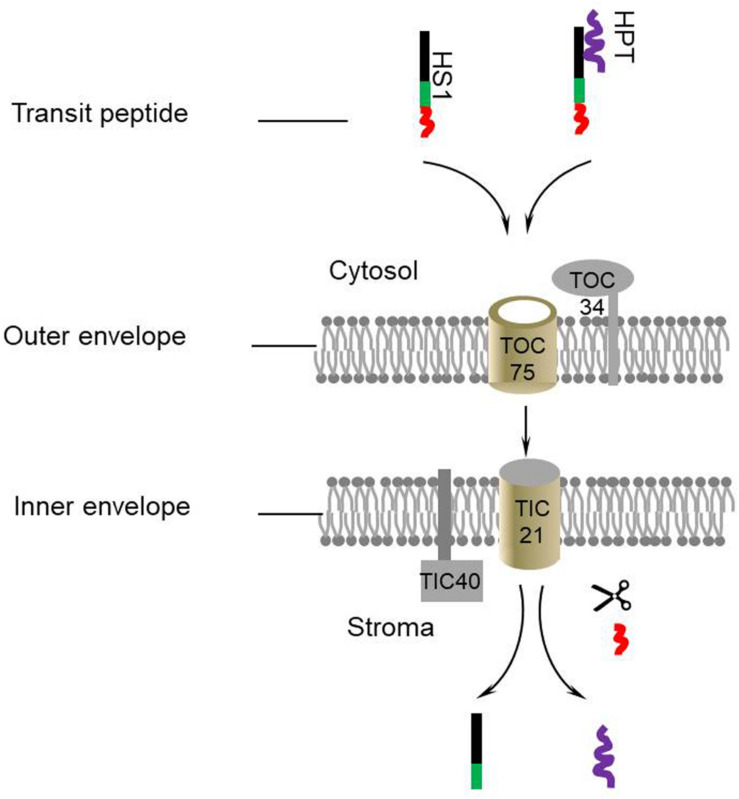
A proposed model for HS1-mediated chloroplast transport of HPT. HS1 directly interacts with HPT and guides the protein to the TOC/TIC complex. Subsequently they are transported into the chloroplast. The transit peptide of HS1 is removed by a stromal processing protease and transit HPT are discharged.

In this work, we demonstrated HS1 is involved in HyB resistance through facilitating the transportation of foreign HPT from the cytosol to chloroplast in Arabidopsis. Based on these results and deductions, a model for HS1-mediated transport of HPT was proposed (Figure 5). HS1 interacts with HPT to form a transport complex and co-transports across envelope membranes of chloroplast into stroma through the classical import pathway. Once imported into the chloroplast, the bound HPT is discharged from HS1. Thus, HS1 acts as a guiding factor (or leading factor) in the process of transporting HPT into chloroplasts. This finding extends beyond the model of the single protein transport machinery that transports transit peptide proteins across the chloroplast membrane and provides novel insights into chloroplast protein import pathways.

This study has enhanced our understanding of antibiotic resistance in transgenic plants. As a positive selective marker of transgenic plants, HPT can phosphorylate and inactivate HyB. Previous reports showed that HPT is present in both intracellular and extracellular spaces of plants and can even diffuse into the medium (Zhang et al., 2011). These studies imply that transgenic plants have the potential to complete the detoxification of HyB before HyB can enter into the chloroplast and target to the 30S subunit of ribosomes. Furthermore, a little amount of HPT still accumulates in chloroplasts of *hs1-1* mutants (Figures 3C,E–H). This finding suggests that HS1 may influence the transport efficiency of HPT. Other factors may be involved in the process which will be explored through additional experiments. Our results show that in the absence of HS1, transgenic plants are hypersensitive to HyB despite high-level HPT expression, indicating the importance of chloroplast importation of HPT in mitigating its toxicity of HyB.

Homologs of HS1 can be found in higher plants where the cTP regions are highly conserved (Supplementary Figures S4, S5). This implies that the HS1-mediated transportation may be a conserved pathway for nucleus-encoded chloroplast cTP-less proteins. HS1-mediated protein transportation, as found in this study, may be an optimal approach for subcellular targeting of many proteins because HS1 provides a flexible, signal peptide-independent pathway for proteins distributed in cells.

In conclusion, our findings show that HPT, a transit sequence-less protein, was imported into chloroplasts in association with HS1. The “piggy-backing” pattern is a previously unidentified aspect of chloroplast protein import. These results suggest that the function of HS1 is to guide cTP-less nucleus-encoded proteins co-transporting into chloroplasts, which beyond the current recognition about a single cTP protein transporting machinery and provides novel insights into chloroplast proteins transport mechanism.

## Data Availability Statement

All datasets generated for this study are included in the article/Supplementary Material.

## Author Contributions

YL and LW conceived and designed the research. YL, LZ, WL, MX, and CZ performed the experiments, analyzed the data. YL drafted the manuscript. LW and CZ contributed to revisions of the manuscript. LW supervised the project. All authors reviewed the manuscript.

## Conflict of Interest

The authors declare that the research was conducted in the absence of any commercial or financial relationships that could be construed as a potential conflict of interest.

## Footnotes

1 http://www.ncbi.nlm.nih.gov/BLAST
