# Supplementary material for: HS1 Is Involved in Hygromycin Resistance Through Facilitating Hygromycin Phosphotransferase Transportation From Cytosol to Chloroplast
Source: Front Plant Sci. 2020 May 27;11:613. doi: 10.3389/fpls.2020.00613 (PMC7266939; doi:10.3389/fpls.2020.00613)
Supplement: Supplementary file 1 [file Data_Sheet_1.docx]

HS1 is involved in hygromycin resistance through facilitating hygromycin phosphotransferase transportation from cytosol to chloroplast

Yanzhong Luo, Lan Zhang, Weiwei Li, Miaoyun Xu, Chunyi Zhang, Lei Wang*

Biotechnology Research Institute, Chinese Academy of Agricultural Sciences, Beijing, 100081, People’s Republic of China

*Author for correspondence: *Lei Wang*

*Email:wanglei01@caas.cn*

**SUPPORTING INFORMATION**

Figure S1. Characterization of Arabidopsis RNAi mutants library.

Figure S2. Phenotypes of the re-transformed *HS1*RNAi lines.

Figure S3. Genotype of *hs1-1* and *hs1-2* mutants.

Figure S4. Alignment of HS1 with its putative homologs from various plant species.

Figure S5. Phylogenetic tree of HS1 homologs proteins in plants.

Figure S6. Immunological estimation of contamination of isolated chloroplasts.

Table S1. Nucleotide sequences of primers used in this study.

Table S2. The insertion sites of constructs and primers used for PCR.


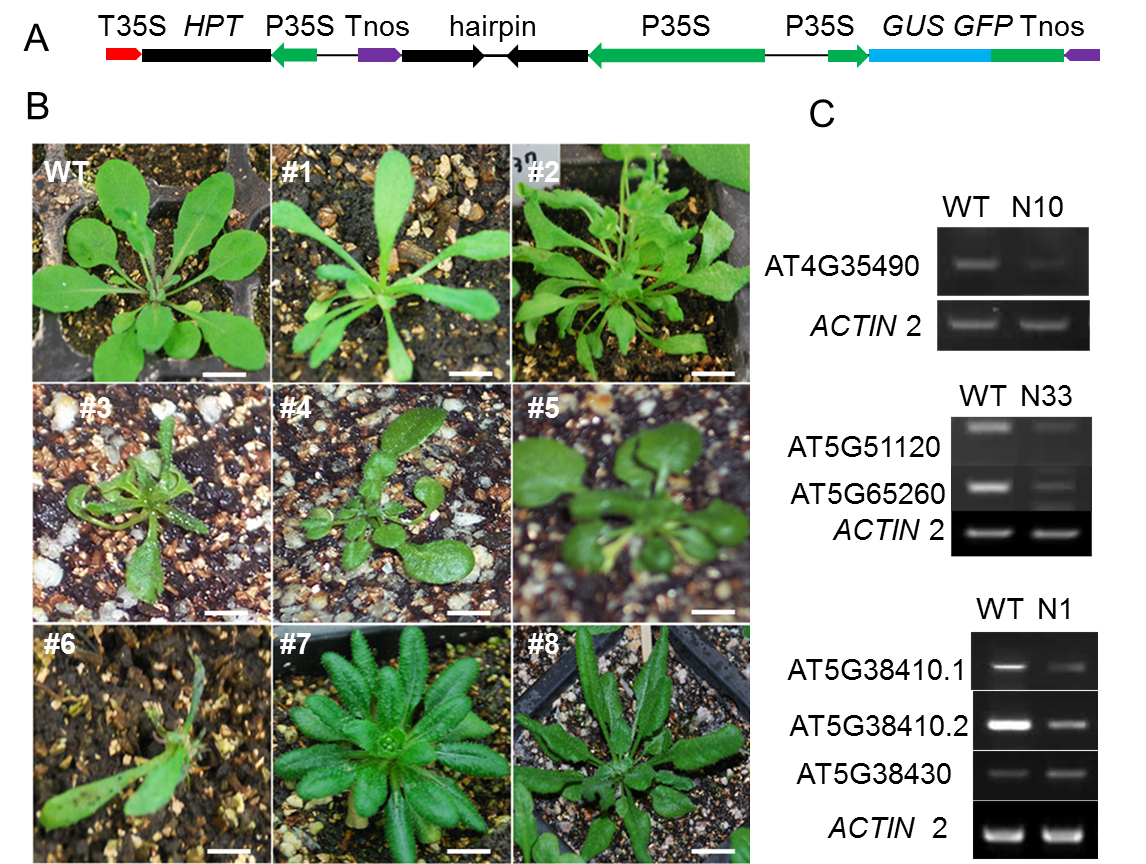


**FIGURE S1. Characterization of Arabidopsis RNAi mutants library.** A: Schematic construct of Arabidopsis RNAi library. B: RNAi mutants display various phenotypes. #1-#8 are different 5 week-old RNAi mutant lines, Bar=1cm. C: Semi-quantitative RT-PCR confirmed the silencing of predicted target genes in each of the Arabidopsis hpRNA lines.


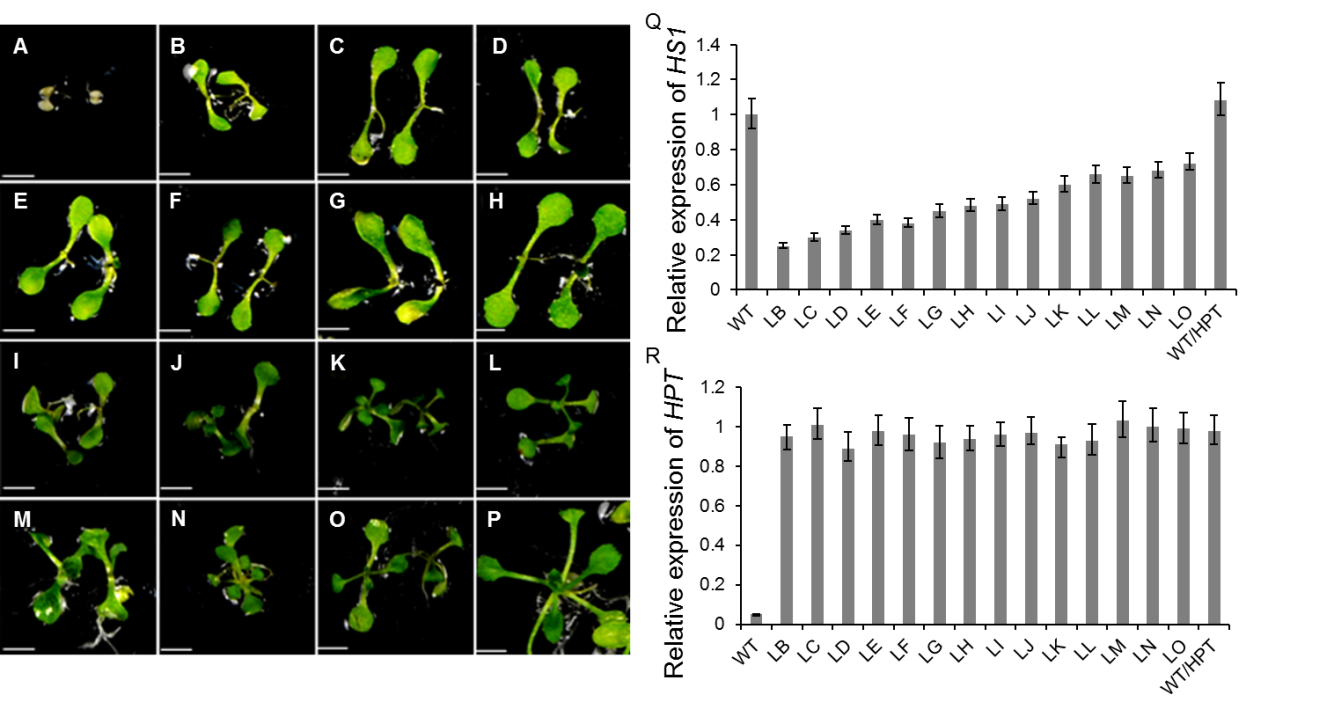


**FIGURE S2. Phenotypes of the re-transformed HS1RNAi lines.** A: WT; B-O: *HS1* RNAi mutant lines. P: CK (HPT harboured empty vector transgenic plant), 10 days seedling. T2 seeds were screened on 0.5╳MS medium supplied with 25mg/L hygromycin. Bars=3mm. Q,R: Quantitative PCR expression analysis of *HS1* (Q) and *HPT* (R) using *ACTIN* as reference.L1-L14 are different hpRNA transformed lines, WT and WT/*HPT* served as control. Plants used for expression analysis grew on0.5╳MS medium without hygromycin supplied.


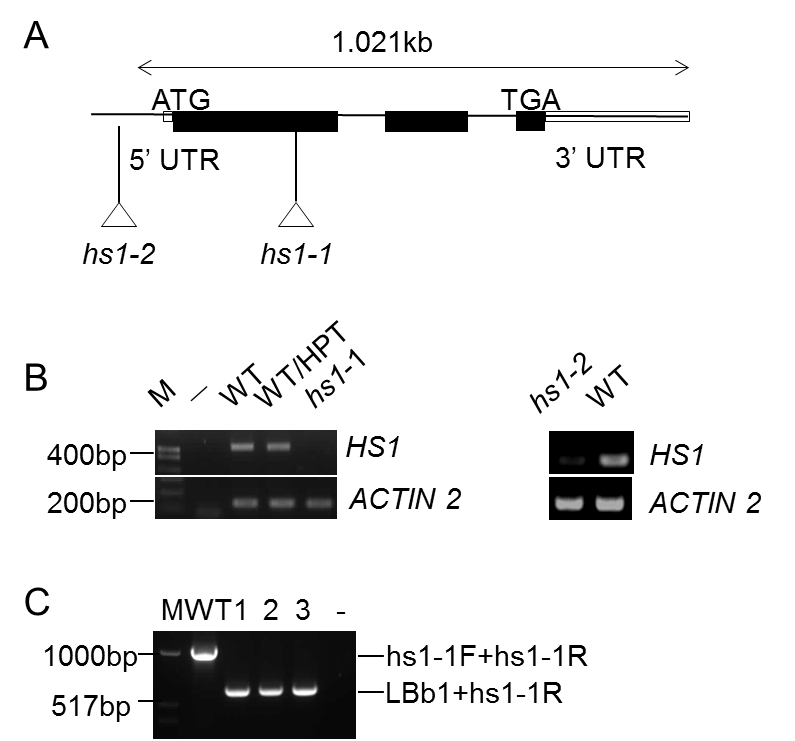


**FIGURE S3. Genotypes of *hs1-1* and *hs1-2* mutants.** A: Schematic structure of T-DNA insertion lines *hs1-1* and *hs1-2*. Black boxes: exons. Triangle: T-DNA insertion site. B: Analysis of *HS1* expression in *hs1-1* and *hs1-2* mutants using semi-quantitative RT-PCR. C: PCR confirmation of *hs1-1* homozygosity (lanes 1, 2 and 3). WT and water (-) served as the positive and negative controls, respectively. M: DNA size marker.


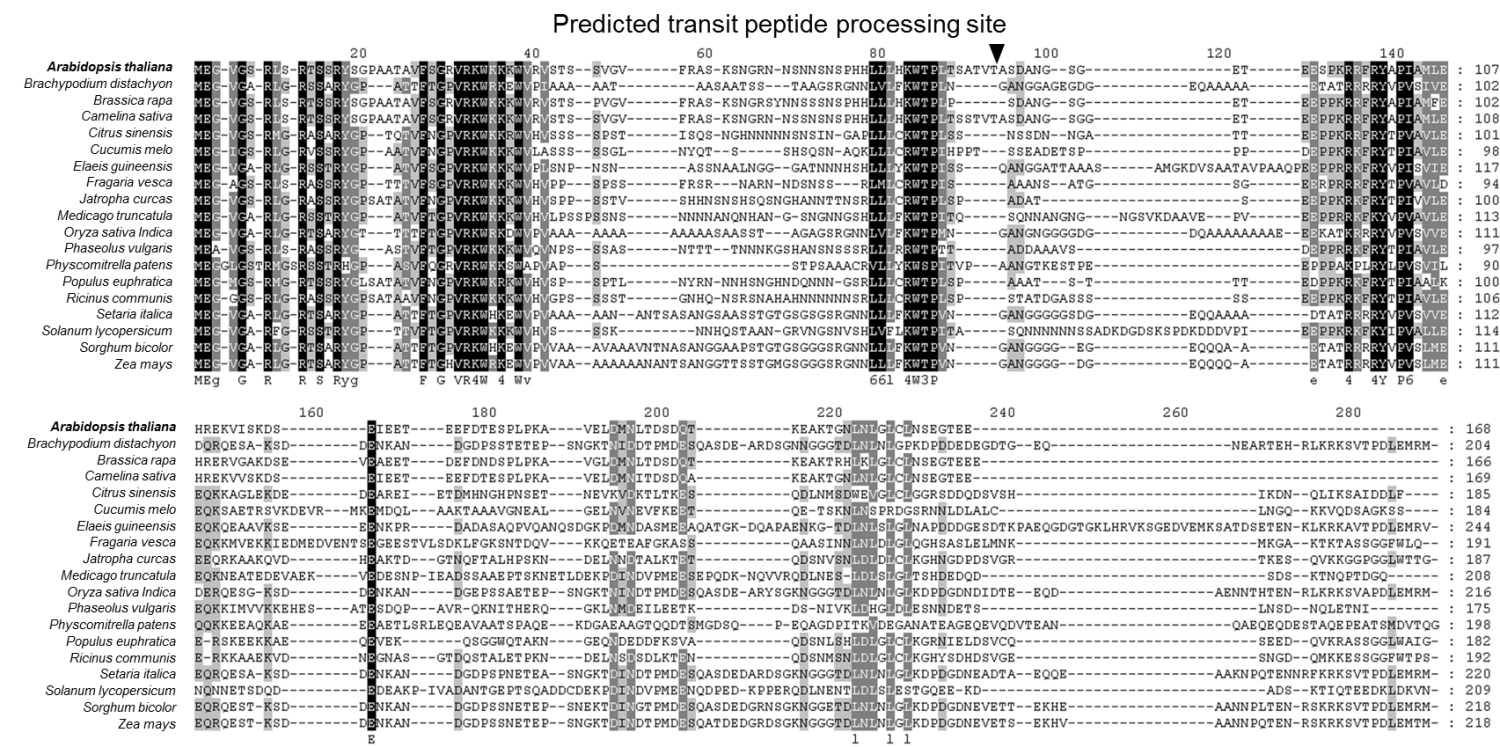


**FIGURE S4. Alignment of HS1 with its putative homologs from various plant species.** A: Alignment of HS1 protein sequences from various plant species were aligned using the Clustal W program. The three-level conservation shading was accomplished using the GeneDoc program. Residues conserved across all sequences are shaded in black; moderately conserved residues are shaded in dark gray or light gray. The numbers 4, 5 and 6 indicate the conserved amino acid groups: 4 = KR (basic), 5=FY (aromatic) and 6 = LIVM (aliphatic or M).The predicted cTP motif region as indicated in top. The arrowheads indicate the classical amino acids of Tat. B: Phylogenetic analysis of HS1 proteins from different plant species. Multiple sequence alignment of the protein sequences was performed using Clustal W. Branch length represents substitutions per site. Accession numbers are as follows: Accession numbers are as follows: *Solanum lycopersicum* (XP_004250252), *Medicago truncatula* (XP_003594196), *Arabidopsis thaliana* (NP_200332), *Camelina sativa* (XP_010449262), *Brassica rapa* (XP_009120042), *Populus euphratica* (XP_011013131), *Cucumis melo* (XP_008447711), *Zea mays* (NP_001143916), *Sorghum bicolor* (XP_002444180), *Setaria italica* (XP_004973235), *Brachypodium distachyon* (XP_003573802), *Oryza sativa* Indica Group (EAZ06615), *Oryza sativa* Japonica Group (BAG98631), and *Physcomitrella patens* (XP_001771058).


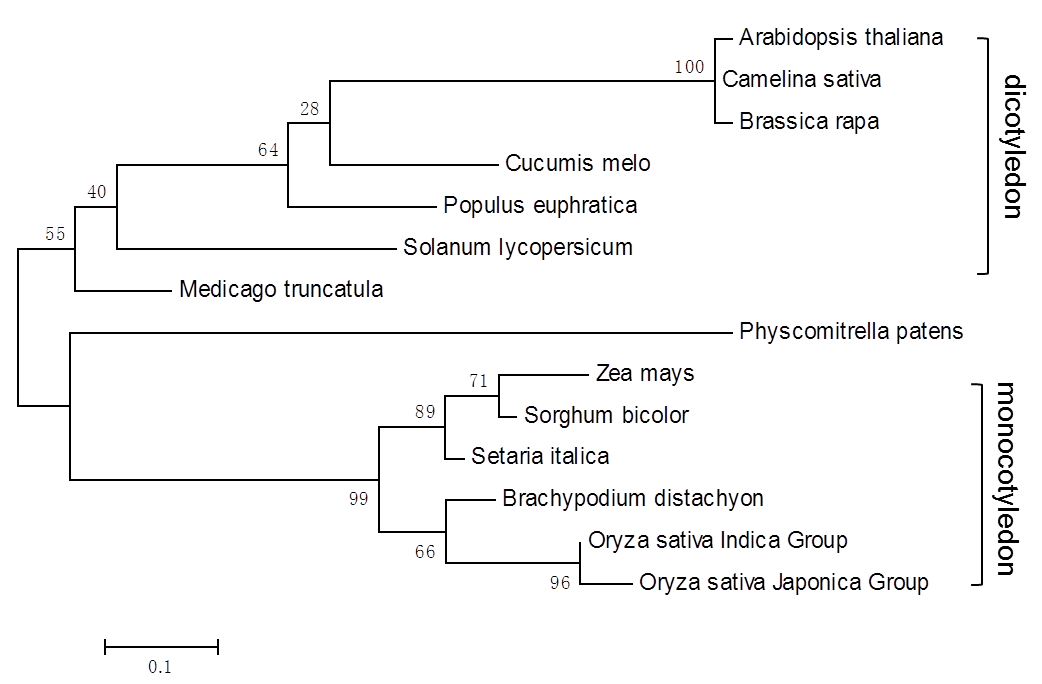


**FIGURE S5. Phylogenetic tree of HS1 homologs proteins in plants.** BlastP was done using the HS1 protein sequence against plants proteins at GenBank database. Multiple sequence alignment of the protein sequences was done using Clustalw2. Maximum-likelihood phylogenetic tree was drawn by MEGA 6.0 using default parameters with 100 times bootstrapping. Numbers above the branches represent bootstrap support based on 100 bootstrap replicates. Branch length represents substitutions per site. GenBank protein accession numbers as Figure S4.


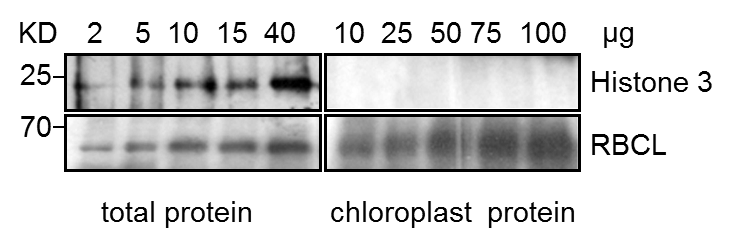


**FIGURE S6. Immunological estimation of contamination of isolated chloroplasts.** To assess for contamination of chloroplast preparations from other cellular compartments, immunoblot analyses were performed on dilution series of total protein extract and isolated chloroplasts. Both samples were derived from 14-day-old, *hs1-1* Arabidopsis. The indicated amounts of each protein were loaded, and blots were probed with antibodies against RBCL (chloroplast) and Histone 3 (nucleus) as indicated in right. The detection of protein bands partial confirming chloroplasts protein preparations have <1% contamination using this chloroplast isolation protocol. N=3.

**TABLE** **S1. Nucleotide sequences of primers used in this study.**

| Name | Nucleotide sequences of primers (5′→3′) |
| --- | --- |
| LBb1 | GCGTGGACCGCTTGCTGCAACT |
| Inrv4: | TCAGACTTTGCAATATGCAGAGCT |
| P35S | CACAATCCCACTATCCTTCG |
| hhs1-1F | ACACAAGACATGTCCAAAGGC |
| hhs1-1R | CAGTTTTTGCTTCCTGCAGAG |
| hhs1-2F | TCAACAGCAAGCATTACATGG |
| hhs1-2R | TAACCGTCGCTGAGGTAAGTG |
| HHS1RTF | ATGGAAGGTGTAGGATCGAGA |
| HHS1RTR | TCTTCGCTTAGGCGACTCCTC |
| HPTRTF | ATGAAAAAGCCTGAACTCACC |
| HPTRTR | GATCGCATCCATAGCCTCCGC |
| HHS1F | ATCTAGAATGGAAGGTGTAGGATCGAGAT |
| HHS1R | AGAGCTCTCATTCTTCAGTCCCTTCAGA |
| HHS1GFPR | ACCATGGCTTCTTCAGTCCCTTCAGA |
| HHS1RFF | CCTCATCCGTCGGTGTTTTCAGA |
| HPTF | CTCTAGAATGAAAAAGCCTGAACTCACC |
| HPTGFPR | TCCATGGATTTCTTTGCCCTCGGACGAG |
| GFPF | ATCTAGAGGTACCATGCACCATCATCATCATCATT |
| GFPR | TGAGCTCTCACACGTGGTGGTGGTGGTGG |
| NGFPF | CGGATCCGTCGACATGGTAGATCTGACTAGTAAA |
| NGFPR | CGAGCTCGTCTTCGATGTTGTGGCGGGTC |
| CGFPF | CGGATCCGTCGACAAGCAAAAGAACGGCATCAAA |
| CGFPR | GAGCTCTCACACGTGGTGGTGGTGGTG |
| HHS1Y2F | ACATATGATGGAAGGTGTAGGATCGAGA |
| HHS1Y2F1 | ACATATGACGGCGGTGTTCAGTGGCCGT |
| HHS1Y2F2 | ACATATGTCATCCGTCGGTGTTTTCAGA |
| HHS1Y2F3 | ACATATGAACTCACCACACCATCTACTT |
| HHS1Y2F4 | ACATATGGCCTCCGACGCTAATGGTTCC |
| HHS1Y2R | CGGATCCTTCTTCAGTCCCTTCAGAATT |
| HPTY2F | GTTAAGATGAAAAAGCCTGAACTCACC |
| HPTY2R | CGGATCCTTTCTTTGCCCTCGGACGAGT |
| TOC75Y2F | GATTACGCTCATATGATGGCCGCCTTCTCCGTCAAC |
| TOC75Y2R | TGCAGCTCGAGCTCGATACCTCTCTCCAAATCGGAA |
| TIC40Y2F | GATTACGCTCATATGATGGAGAACCTTACCCTAGTT |
| TIC40Y2R | TGCAGCTCGAGCTCGACCCGTCATTCCTGGGAAGAG |
| TOC34Y2F | GATTACGCTCATATGATGGCAGCTTTGCAAACGCTT |
| TOC34Y2R | TGCAGCTCGAGCTCGAGACCTTCGACTTGCTAAACC |
| TIC21Y2F | GATTACGCTCATATGATGCAATCACTACTCTTGCCG |
| TIC21Y2R | TGCAGCTCGAGCTCGAGCAACCTTAGGAACTACGAC |

These primer pairs were used as gene specific primers in PCR analysis or mutants identification. F is the forward and R the reverse primer.

**TABLE** **S2. The insertion sites of constructs and primers used for PCR**

| Notation in article | Backbone of vector | Insertion sites | Primers |
| --- | --- | --- | --- |
| WT/HPT | pCAMBIA1303 |  |  |
| HS1-1/HPT | pCAMBIA1303 |  |  |
| WT/HPT/HS1 | pCAMBIA1303-GUS | Xba I-Sac I | HS1F, HS1R |
| HS1-1/HPT/HS1 | pCAMBIA1303-GUS | Xba I-Sac I | HS1F, HS1R |
| pHS1-HS1GFP | pCAMBIA1303 | Hind III-Nco I | HS1F, HS1GFPR;P45F, P45R |
| p35S-GFP | pCAMBIA1303 |  |  |
| HS1RNAi | pCAMBIA1303-GUS | Xba I-sac I | HS1RiF, HS1RiR |
| HPT-YFP | pSP72-YFP | Xba I-Nco I | HPTF, HPTGFPR |
| HS1-GFP | pSP72-GFP | Xba I-Nco I | HS1F, HS1GFPR |
| HPT-GFP | pSP72-GFP | Xba I-Nco I | HPTF, HPTGFPR |
| NGFP | pSP72-GFP | Xba I-Nco I | NGFPF, NGFPR |
| CGFP | pSP72-GFP | Xba I-Nco I | CGFPF, CGFPR |
| NGFP-HS1 | pSP72-GFP | Xba I-Nco I | HS1F, HS1GFPR |
| HPT-CGFP | pSP72-GFP | Xba I-Nco I | HPTF, HPTGFPR |
| BD-HS1F | pGBKT7 | NdeI-EcoRI | HS1Y2F, HSHY2R |
| BD-HS1F1 | pGBKT7 | NdeI-EcoRI | HS1Y2F1, HSHY2R |
| BD-HS1F2 | pGBKT7 | NdeI-EcoRI | HS1Y2F2, HSHY2R |
| BD-HS1F3 | pGBKT7 | NdeI-EcoRI | HS1Y2F3 , HSHY2R |
| BD-HS1F4 | pGBKT7 | NdeI-EcoRI | HS1Y2F4 ,HSHY2R |
| AD-HPT | pGADT7 | Mse I-BamH I | HPTY2F, HPTY2R |
| AD-TOC75 | pGADT7 | EcoR I | TOC75Y2F, TOC75Y2R |
| AD-TOC34 | pGADT7 | EcoR I | TOC34Y2F, TOC34Y2R |
| AD-TIC21 | pGADT7 | EcoR I | TIC21Y2F, TIC21Y2R |
| AD-TIC40 | pGADT7 | EcoR I | TIC40Y2F, TIC40Y2R |

Each full length DNA encode listed proteins were amplified from Arabidopsis cDNA or vector used listed primer pairs and cloned into appropriate vector through insertion sites
